# Impacts of indoor high temperature on cardiovascular health among community-dwelling adults: a scoping review

**DOI:** 10.1136/bmjopen-2026-123980

**Published:** 2026-11-06

**Authors:** Jianying Liao, Nibedita S Ray-Bennett, Joshua Vande Hey, Liu Yang, Samuel Cai

**Affiliations:** 1 Centre for Environmental Health and Sustainability, University of Leicester, Leicester, UK; 2 Institute for Environmental Futures, University of Leicester, Leicester, UK; 3 Avoidable Deaths Network, School of Business, University of Leicester, Leicester, UK; 4 Earth Observation Science Division, School of Physics and Astronomy, University of Leicester, Leicester, UK; 5 School of Public Health, Shanxi Medical University, Taiyuan, China; 6 NIHR Leicester Biomedical Research Centre, Leicester, UK; 7 The NIHR Health Protection Research Unit (HPRU) in Chemical Threats and Hazards at the University of Leicester, Leicester, UK; 8 Leicester British Heart Foundation Centre of Research Excellence, University of Leicester British Heart Foundation Cardiovascular Research Centre, Leicester, UK

**Keywords:** Cardiovascular Disease, Blood Pressure, PUBLIC HEALTH, EPIDEMIOLOGY

## Abstract

**Abstract:**

**Objectives:**

To map and summarise evidence on the association between indoor high temperature exposure and cardiovascular health outcomes among community-dwelling adults.

**Design:**

Scoping review.

**Data sources:**

MEDLINE, PubMed and Web of Science were searched in March 2026 for studies published between 1 January 2014 and 31 December 2025.

**Eligibility criteria:**

Studies were included if they involved community-dwelling adults, objectively measured or reported indoor temperature and examined cardiovascular outcomes including blood pressure, cardiovascular symptoms, emergency visits, hospitalisation or mortality.

**Data extraction and synthesis:**

Data were extracted on study design, setting, population, exposure assessment, cardiovascular outcomes, confounders and key findings. Findings were summarised narratively.

**Results:**

A total of 18 studies were included. Most studies were conducted in Asia (n=12, 66.7%), followed by North America (n=4, 22.2%), Europe (n=1, 5.6%) and Australia (n=1, 5.6%). Cross-sectional studies were the most common design (n=10, 55.6%), followed by cohort, panel, case–control and experimental studies (each n=2, 11.1%). Blood pressure or blood pressure-related outcomes were the most frequently examined outcomes (n=13, 72.2%), whereas fewer studies examined other haemodynamic measures, cardiac autonomic responses, heat-related symptoms or clinical cardiovascular morbidity and events. Exposure assessment methods varied substantially, with most studies using direct indoor temperature monitoring in participants’ homes (n=12, 66.7%).

**Conclusions:**

Current evidence remains limited and heterogeneous, particularly for heat-related cardiovascular outcomes beyond blood pressure. Future studies with robust indoor exposure assessment, incorporating housing, behavioural and environmental factors are warranted to investigate a wide range of cardiovascular outcomes.

**STRENGTHS AND LIMITATIONS OF THIS STUDY**

- This scoping review systematically mapped recent evidence on indoor temperature exposure and cardiovascular health among community-dwelling adults.
- The review followed Joanna Briggs Institute methodology and was reported in accordance with Preferred Reporting Items for Systematic Reviews and Meta-Analyses extension for Scoping Reviews (PRISMA-ScR).
- Detailed information was extracted on study design, population characteristics, exposure assessment methods, cardiovascular outcomes, confounder adjustment and key findings.
- Only English-language peer-reviewed studies were included, and grey literature was not searched.
- A formal quality assessment was not undertaken, which may limit interpretation of the strength and certainty of evidence from individual studies.

## Introduction

 As the climate changes, the frequency and intensity of heatwaves (ie, periods of high temperature) have been increasing over the years^1–5^ globally. The year of 2024 was the warmest year on record^6^ with the record being projected to be broken under enhanced global warming in future years. The rising temperature has heightened health risks, particularly among those with chronic health conditions such as cardiovascular diseases (CVDs). Studies have shown that each 1°C increase in outdoor temperature correlates with a 2.1% rise in CVD-related deaths, with such risk increasing by 11.7% during heatwaves.^7^ The serious health impacts of outdoor heat stress on hospitalisations and premature deaths are well documented.^8–11^ While much scientific endeavour has been focused on health impacts from outdoor heat, people worldwide spend approximately 80–90% of their time indoors.^12–14^ Indoor environments can potentially exacerbate heat exposure, especially among those with limited capacities to manage high temperatures. Factors such as age, pre-existing health conditions and personal behaviours influence how individuals respond to indoor over-heating. This is particularly concerning for those with CVDs, as heat exposure can aggravate symptoms and heighten hospitalisation risks. Recognising the potential health hazards of indoor heat, the WHO has called for measures to mitigate indoor over-heating as a component of heat-health action plans.^15 16^

In the UK, summer heatwave events in 2026 have highlighted the need for evidence-based guidelines that address indoor high temperature, especially since the current guidelines largely rely on outdoor temperature data.^17^ This approach does not account for the unique factors influencing indoor temperature, such as neighbourhood design, building materials and individual behaviours such as window opening and using fans or air-conditioning. Additionally, there is no established threshold for indoor temperatures in residential settings to safeguard health, particularly for those with heat-sensitive health conditions. In several North American and European cities, between 54% and 98% of heat-related deaths have been reported to occur indoors,^18^ underscoring the need for tailored guidance that addresses indoor over-heating risks on health.

Although there is currently no comprehensive review specifically on the effects of indoor temperature on CVD, a 2017 systematic review and meta-analysis^19^ did examine the relationship between environmental temperature and blood pressure (BP), focusing on indoor conditions. It found that a 1°C decrease in indoor temperature led to a 0.38 mm Hg (95% CI 0.18 to 0.58) increase in systolic BP, especially affecting individuals with CVD who are more sensitive to temperature changes. Another recent meta-analysis^20^ on 22 studies published up to 2020 examined the impact of high indoor temperatures on various health outcomes, with a particular focus on vulnerable populations. The review found some evidence of adverse impacts from high indoor temperatures on respiratory health, diabetes management, core schizophrenia and dementia symptoms. However, it did not identify studies on patients with manifested CVD outcomes (other than BP from six studies) that are known to be heat sensitive. Therefore, there remains a significant gap in understanding how various ranges of indoor temperature specifically impact CVD outcomes across different populations and settings. This scoping review aimed to map and summarise recent evidence on indoor temperature exposure and cardiovascular health among community-dwelling adults. Specifically, it sought to describe study characteristics, exposure assessment methods, cardiovascular outcomes and key methodological gaps to inform future research and heat-health guidance.

## Methods

This scoping review was conducted following the Joanna Briggs Institute methodology for scoping reviews^21^ and reported in accordance with the Preferred Reporting Items for Systematic Reviews and Meta-Analyses extension for Scoping Reviews (PRISMA-ScR).^22^ A completed PRISMA-ScR checklist is provided in online supplemental file 3. No formal protocol was registered for this scoping review. However, the review question, eligibility criteria, search strategy and data extraction framework were developed before screening.

### Identifying the research question and inclusion criteria

The research question and the inclusion/exclusion criteria are shown in table 1. We identified studies published from 1 January 2014 to 31 December 2025. This time frame ensured a focus on recent literature to inform future research priorities. No specific exclusion criteria were set based on geographical location, ethnicity or biological sex group, enabling identification of a broad range of potential studies. Eligible study designs included randomised controlled trials, non-randomised controlled trials, prospective and retrospective cohort studies, case–control studies and cross-sectional studies.

**Table 1:** Research question and inclusion/exclusion criteria

PECO element	Description
Research question	How does indoor temperature affect cardiovascular health among community-dwelling adults, including hypertension, cardiovascular symptom reporting, hospitalisation and mortality?
Population	Community-dwelling adults
Exposure	Indoor temperature
Comparator/association criteria	Risk estimates adjusted for at least age and sex; two or more exposure groups or provision of a linear trend.
Outcome	Cardiovascular health outcomes, including hypertension, blood pressure, cardiovascular symptom reporting, hospitalisation and mortality.
Exclusion	Studies published before 1 January 2014. Studies focusing exclusively on children (<18 years old) or adults in residential care or hospitalised populations. Studies where indoor temperature is not explicitly measured or reported. Studies not published in English.

### Search strategy

This scoping review undertook a comprehensive literature search across several major databases, including MEDLINE, PubMed and Web of Science, to identify studies relevant to indoor temperature exposure and CVDs. A combination of Medical Subject Headings and free-text terms was employed to ensure thoroughness and relevance. Primary search terms encompassed “indoor high temperature” or “indoor heat exposure”, along with terms such as “cardiovascular diseases”, “heart disease”, “hypertension” and “high blood pressure”. Boolean operators (AND, OR) were used to structure the search queries effectively for each database. The final search was conducted March 2026 (online supplemental file 1A).

### Evidence screening, selection and data extraction

Following the search, all identified studies were collated and uploaded into EndNote V.20 (Clarivate Analytics, Pennsylvania, USA), and duplicates were removed. Titles and abstracts were screened against the eligibility criteria, followed by full-text assessment of potentially relevant studies. Reasons for exclusion at the full-text stage were recorded and are presented in the PRISMA flow diagram.

Data were extracted from the included studies by the lead author using a structured data extraction form. Extracted variables included author, year of publication, research location, study design, study population, sample size, indoor temperature assessment method, assessment period, exposure range, cardiovascular health outcome, confounders and main findings. Uncertainties during screening or data extraction were discussed with the supervisory team.

### Data synthesis

Findings were synthesised narratively. Included studies were summarised according to study characteristics, geographical region, population, study design, cardiovascular outcome, indoor temperature exposure assessment and key methodological features. Meta-analysis was not undertaken because of heterogeneity in study designs, exposure metrics, outcome measures and statistical methods.

### Patient and public involvement statement

Patients and members of the public were not involved in the design, conduct, reporting or dissemination plans of this scoping review.

## Results

Database searches identified 686 records. After removing 130 duplicates, 556 records were screened by title and abstract, 42 full-text articles were assessed for eligibility and 18 studies were included in this scoping review. The full list of included studies is provided in online supplemental file 1B. The study selection process is presented in figure 1.

**Figure 1:**
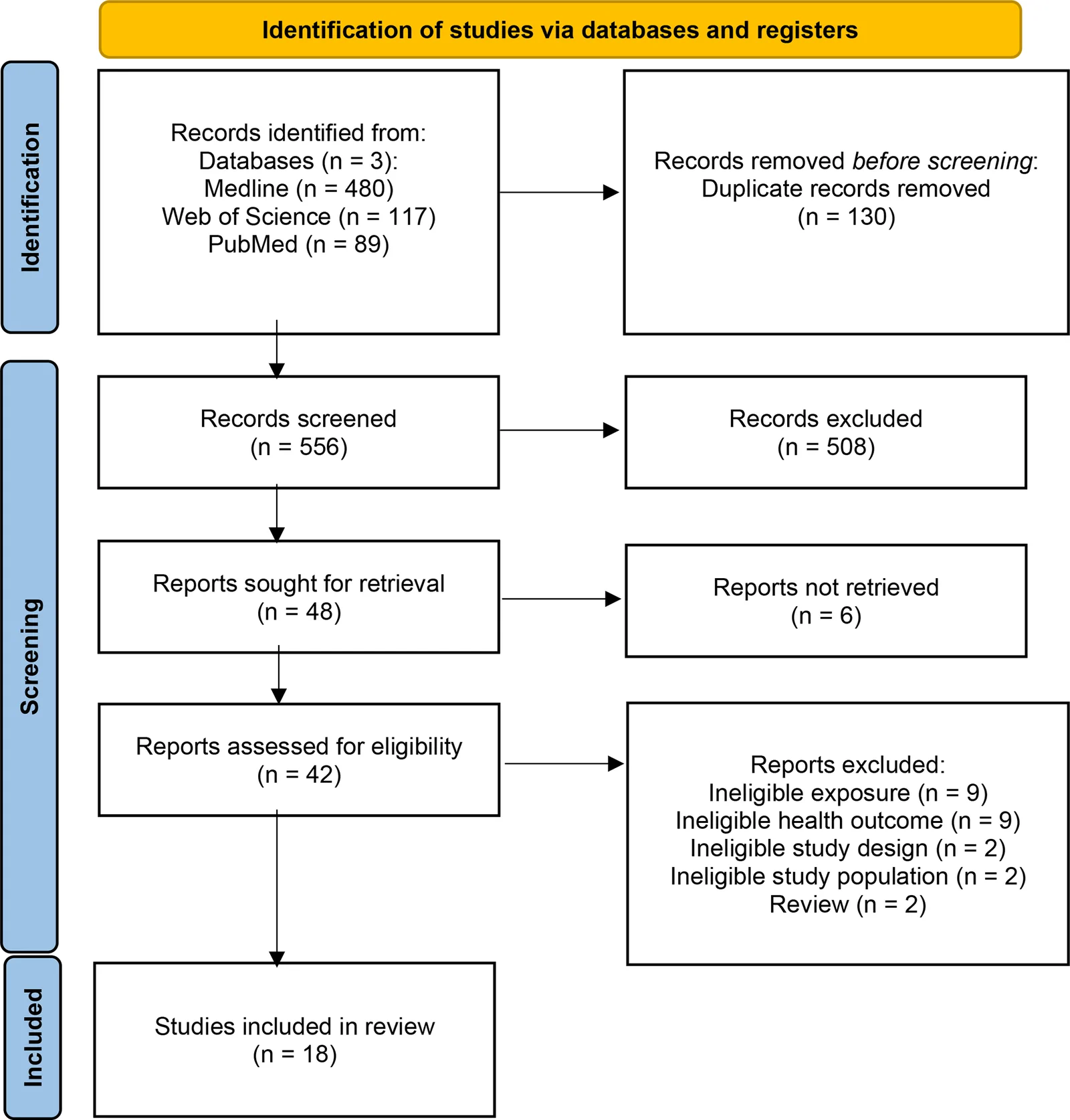
PRISMA flow diagram of study identification, screening, eligibility assessment and inclusion. PRISMA, Preferred Reporting Items for Systematic Reviews and Meta-Analyses.

### General study characteristics

The included studies were published between 2014 and 2025, with more studies published after 2020. They were conducted across seven countries or regions, with most conducted in Asia (n=12, 66.7%). Cross-sectional studies were the most common design (n=10, 55.6%). Other study designs included cohort (n=2, 11.1%), panel (n=2, 11.1%), case–control (n=2, 11.1%) and experimental studies (n=2, 11.1%). Full study characteristics, including study design, region, target population, participant characteristics, health outcomes, confounders and key findings, are presented in online supplemental file 2, table S1.

### Characteristics of the study populations

Sample sizes varied widely across the included studies, ranging from 16 to 260 465 participants. 11 studies (61.1%) included fewer than 500 participants, with sample sizes ranging from 16 to 401.^23–33^ Among these, five studies had particularly small sample sizes of 55,^24^ 48,^26^ 47,^33^ 46^29^ and 16.^32^ Among the rest, a cross-sectional study in Taiwan^34^ included 260 465 participants, while others ranged from 735 to 4659.^35–40^

Regarding age, half of the studies (N=9, 50%)^23–25 28 29 32–34 39^ focused on individuals aged over 60 years, followed by 50–60 years (N=5, 27.8%)^30 35 36 38 40^ and under 50 years (N=4, 22.2%).^26 30 31 37^ Average age ranged from 36.3 to 79.6 years.

Sex distribution was generally balanced in most studies (N=11, 61%)^23 29–31 34–40^ with male representation between 44.1% and 56.0%, though five studies^25–28 33^ reported less balanced distributions (27.9–35.4% of male participants). One heart failure study^24^ had a male proportion of 90.9%.

Hypertension was the most studied long-term CVD, with eight studies^23 25 28 30 36–39^ reporting prevalence or antihypertensive use (21.6–57.7%). One study in China^29^ did not report hypertension prevalence but showed elevated systolic blood pressure (SBP) (135.94±19.36 mm Hg). Two studies in New York^35 40^ focused on distress call for cardiac arrest, cardiac condition and other potential cardiovascular cases. Another study in Taiwan^34^ focused on CVDs emergency department visits. One study focused on individuals with heart failure.^24^ Four studies^26 27 32 33^ recruited healthy participants.

### Exposure assessment

The exposure assessment characteristics are summarised in online supplemental file 2, table S2. All studies objectively measured indoor temperature, with most (66.7%, N=12)^23–25 27–30 33 36–39^ conducted in participants’ homes, and others in exposure controlled rooms,^31 32^ settings where patients received emergency care,^35 40^ unspecified indoor settings^26^ and one by data modelling.^34^ Nearly half (44.4%, N=8)^23 27 29 33 36–39^ of the included studies conducted measurements in living rooms or bedrooms, placing instruments 0.6–1.5 m high and away from heat sources like radiators or sunlight.

#### Monitoring campaign

13 studies used indoor temperature monitor in their research. Among these, six studies^23 24 27 33 36 38^ monitored indoor temperature at 10-minute time resolution for varying monitoring periods from hours to months. One study^29^ monitored indoor temperature at 1-minute time resolution for 4 months. Three studies monitored indoor temperature for two times per day, continuously for 18 days,^25^ 1-year^28^ and 2-year.^30^ One study^26^ monitored indoor temperature at bi-weekly interval for 2 years. And one study did not specify the time resolution but monitored for 1 week.^39^ Finally, a snapshot temperature measurement at a single time point was reported in one study from England.^37^

The remaining five studies, two controlled exposure studies implemented laboratory-based monitoring campaigns under tightly regulated thermal conditions. One study applied rapid temperature transitions of ±10°C (18–28°C or vice versa) during a short-term 20-minute exposure^31^ whereas another conducted prolonged daylong exposure sessions lasting approximately 8 hours under fixed indoor temperature conditions, with continuous physiological monitoring throughout^32^; two used portable monitor which monitored indoor temperature at 2-minute or 3-minute time interval for an uncertain short-term period, depends on the time paramedics spent on site and sensor response time requirements^35 40^; one was a modelling study in which indoor temperature estimates were calculated from a model.^34^

#### Calibration and validation

Calibration or validation of instruments was detailed in six of the included studies. The Canadian study^24^ monitored device performance through weekly data downloads, zero-point checks and cleaning of impaction plates. A 2018 South Korean heatwave study^25^ deployed 20 devices in the same indoor space, recording temperature every 15 min over 40 min, with adjustments based on Wet Bulb Globe Temperature levels using the QUESTemp°30 device. Kubozono *et al*^28^ validated temperature readings by comparing their devices with a standard thermometer, altering the temperature 20 times for accuracy. Another study^30^ used the same device as Kubozono *et al* did, citing its prior validation for reliability. O’Connor *et al*^33^ reported sensor accuracy (±0.1°C) and applied data quality control procedures including artefact removal, exclusion thresholds and time-aligned processing with physiological measurements. Gonsoroski *et al*^40^ applied data validation methods including cross-sensor comparison and removal of anomalous readings.

#### Exposures combined with individual time-activity data or housing characteristics

33.3% (N=6) of the studies integrated environmental exposure with individual behaviour or housing characteristics. Three studies^23 38 39^ reported the time participants spent at home and outdoors. Saeki *et al*^23^ found ambient temperature (combining indoor and outdoor exposure) outperformed indoor temperature in data analysis, with better Akaike information criterion values for sleep-through morning BP surge (6094 vs 6245) and pre-waking BP surge (6057 vs 6212). Umishio *et al*^38^ reported participants spent 17.3 hours daily at home (72% of their time), while Song *et al*^39^ observed older adults spent more time indoors, though in some regions, over half spent half their day outdoors.

Goldberg *et al*^24^ included ‘window use’ and ‘air conditioning’ as covariates in a mixed-effects regression model linking oxygen saturation, BP and temperature. Umishio *et al*^36^ recorded wake-up times and corresponding indoor temperatures using daily diaries instead of fixed points. Two studies reported housing characteristics. A study in Taiwan^34^ included housing characteristics in the model for predicting indoor temperature. A multicentre study in China^39^ collected housing characteristics and quantified indoor temperature relationships with housing area, heating systems and devices, window type and thermal insulation material.

#### Exposure range

Among all studies reporting the exposure range, the mean estimates of indoor temperature exposure ranged from 10.3°C to 30.5°C. The reported highest average indoor temperature among all studies reviewed was (30.5±2.2°C), recorded during the 2018 Northeast Asia heatwave in South Korea.^25^ Another summer indoor temperature assessment conducted in New York, USA,^35^ reported relatively moderate values, with a daytime mean of 23.9±2.5°C (range: 13.5–33.5°C) and a night-time mean of 22.9±2.5°C (range: 12.8–29.3°C). The lowest average indoor temperature was measured during winter in Nara, Japan. Saeki *et al*^23^ measured winter morning temperatures in bedrooms and living rooms, with the lowest average of (10.3±4)°C recorded in bedrooms. In a nationwide study in Japan, bedroom, living room and changing room temperatures were measured, showing similarly low morning winter averages, with the changing room at (10.8±4.1)°C.

Studies in which indoor temperature monitors were not directly deployed included the modelling study in Taiwan,^34^ and two controlled exposure trials conducted in Nanchang, China^31^ and Ottawa, Canada.^32^ The Taiwan study used a mixed-effects model to estimate hourly indoor temperatures, incorporating environmental data, building characteristics, occupant behaviour and electricity use. Cumulative degree hours (CDH) metric was introduced in the study, quantifying temperature exposure during warm (>27°C) and cold (<23°C) seasons and linking it to cardiovascular risk. The Nanchang study exposed participants to controlled temperature fluctuations (±10°C, 18–28°C) over 20-minute intervals, with humidity at 50–60%.

Two New York studies^35 40^ uniquely recorded temperature and humidity at the locations where patients received emergency care, further highlighting the diversity in methodologies.

### Cardiovascular health outcomes

#### Relationships between indoor temperature and cardiovascular health outcomes

Among the reviewed studies, 13 (72.2%) identified BP as the main health outcome, with 12 consistently reporting a significant inverse relationship between BP and indoor temperature. Three studies^29 37 39^ linked lower indoor temperatures to higher hypertension morbidity risk. One^34^ associated higher indoor temperature with increased cardiovascular emergency visit; however, two studies^35 40^ found this relationship was not significant. Two studies^27 38^ found a positive correlation between SBP variability and indoor temperature fluctuations, but diastolic blood pressure (DBP) results varied. One study on heart failure^24^ patients reported higher temperatures correlated with shortness of breath and oxygen saturation. Two studies^32 33^ reported significant increases in heart rate and reductions in heart rate variability under high indoor temperature conditions. Details on statistical significance and effect direction are in table 2.

**Table 2:** Statistical significance and direction of associations between indoor temperature exposures and outcomes

Health outcomes	Statistical significance			
	Significant (+)	Non-significant (+)	Significant (−)	Non-significant (−)
SBP			Zhao *et al*^37^; Chen *et al*^31^ 10°C decrease; Song *et al*^39^; Goldberg *et al*^24^; Saeki *et al*^23^; Ni *et al*^26^; Chen *et al*^27^; Li *et al*^29^ age >65 years; Kubozono *et al*^28^; Kinuta *et al*^30^; Umishio *et al*^36^; Meade *et al*^32^ ≥31°C	Kim *et al*^25^ SBP; Chen *et al*^31^ 3°C increase; Li *et al*^29^ age ≤65 years group; Kubozono *et al*^28^ July showed weak, non-significant associations
DBP			Kim *et al*^25^ Hypertension group Chen *et al*^31^ 10°C decrease, normotensive; Zhao *et al*^37^; Song *et al*^39^; Goldberg *et al*^24^; Ni *et al*^26^; Chen *et al*^27^; Li *et al*^29^ age >65 years; Kubozono *et al*^28^; Kinuta *et al*^30^; Umishio *et al*^36^; Meade *et al*^32^ ≥31°C	Chen *et al*^31^ 10°C decrease, hypertensive and 3°C increase; Li *et al*^29^ age ≤65 years group
HR	Meade *et al*^32^; O’Connor *et al*^33^			
HRV			Meade *et al*^32^; O’Connor *et al*^33^	
BPV	Chen *et al*:^27^ SBP; Umishio *et al*:^38^ Day-by-day BPV, diurnal BPV (SBP/DBP)			Chen *et al*:^27^ DBP
CVD emergency visits/calls	Jung *et al*^34^	Uejio *et al*^35^; Gonsoroski *et al*^40^		
Hypertension risk			Zhao *et al*:^37^ Stage 1 and Stage 2 hypertension morbidity risk. Song *et al*^39^; Li *et al*:^29^ age >65 years group hypertension morbidity risk	Li *et al*:^29^ age ≤65 years group
Shortness of breath at night	Goldberg *et al*^24^			
Oxygen saturation	Goldberg *et al*^24^			
Sleep duration				Kim *et al*^25^

BPV, blood pressure variability; CVD, cardiovascular disease; DBP, diastolic blood pressure; HRV, heart rate variability; SBP, systolic blood pressure.

Eight studies examined the relationships between indoor temperature and health outcomes during warm periods. In South Korea,^25^ a 1°C increase in indoor temperature during a 2018 heatwave reduced DBP by 0.44 mm Hg (95% CI −0.84 to −0.04) in older adults with hypertension. Similarly, a year-long study in Japan^28^ found that in August, a 1°C increase in room temperature decreased morning SBP by 0.49 mm Hg (95% CI −0.71 to −0.27) and DBP by 0.27 mm Hg (95% CI −0.39 to −0.15). An experimental study^31^ reported a 10°C increase in indoor temperature reduced SBP by 4.00 mm Hg (95% CI 1.44 to 6.59), with a greater effect being observed among hypertensive participants (7.3 mm Hg vs 1.2 mm Hg in non-hypertensive participants). In a randomised crossover trial^32^ involving four randomised 8-hour indoor exposure sessions at 22°C, 26°C, 31°C and 36°C, SBP was significantly lower at higher temperatures. Compared with 22°C, exposure to 31°C and 36°C reduced SBP by 26 mm Hg (95% CI −35 to −17) and 29 mm Hg (95% CI −38 to −20), respectively, whereas no clinically meaningful change was observed at 26°C.

Other adverse outcomes under heat exposure were also reported. In Taiwan,^34^ cardiovascular emergency visits increased by 14.5%, with a relative risk (RR) of 1.145 (95% CI 1.003 to 1.307), during the warm season when indoor temperatures exceeded 27°C for over 62 hours. In the Canadian randomised crossover trial study,^32^ higher indoor temperatures were also associated with significant cardiovascular strain. Compared with 22°C, exposure to 31°C and 36°C increased heart rate by 9 beats per minute (95% CI 6 to 12) and 13 beats per minute (95% CI 10 to 16), respectively. In addition, heart rate variability declined significantly at temperatures ≥31°C. In Canada,^24^ higher indoor temperatures were associated with reduced SBP (−0.99 mm Hg, 95% CI −2.01 to 0.03), oxygen saturation (−0.14%, 95% CI −0.25 to −0.03), self-rated health (−2.13, 95% CI −3.11 to −1.15) and a worsening in night-time shortness of breath (−2.32, 95% CI −3.67 to −0.98) among patients with heart failure. However, evidence from a study in New York was mixed but generally non-significant for indoor heat exposure. Uejio *et al*^35^ found indoor heat indices above 26°C were associated with respiratory distress by 63% (OR: 1.63, 95% CI 0.98 to 2.68) but showed no significant association with cardiovascular distress (OR: 0.85, 95% CI 0.55 to 1.31). Similarly, Gonsoroski *et al*^40^ observed elevated odds of cardiovascular emergency calls at indoor temperatures ≥28°C (OR: 1.44, 95% CI 0.97 to 2.13), although the association was not statistically significant. In contrast, outdoor temperature was significantly associated with increased cardiovascular risk (OR: 1.12, 95% CI 1.05 to 1.19).

Studies during moderate and cold periods generally found low temperatures associated with increased BP. A cross-sectional study in rural areas^29^ reported that at 13°C, the predicted hypertension prevalence among older adults aged 75 was 56%, rising to 77% at 1.3°C. For every 1°C increase in indoor temperature, the predicted prevalence decreased by 1.9% (95% CI 1.8% to 2.0%) for 75 years old, 2.2% (95% CI 2.0% to 2.4%) for 65 years old, and 2.7% (95% CI 2.5% to 2.9%) for 55 years old. These results illustrate the strong inverse relationship between indoor temperature and hypertension prevalence.

Another key outcome was BP variability due to temperature fluctuations. Chen *et al*^27^ found that morning temperature fluctuations over 1.1°C significantly increased BP variability, with each 1°C rise causing a 2.18 mm Hg increase in SBP SD. Another study^38^ reported that indoor temperature instability increased both diurnal and day-by-day BP variability, with a 1°C rise in morning-evening indoor temperature difference associated with a 0.85 mm Hg increase in SBP and 0.53 mm Hg in DBP.

#### Relationships between indoor and outdoor temperature

13 studies^23–26 28 30 34–40^ included in the review obtained outdoor temperature data during the assessment period. In most studies, outdoor temperatures were recorded as hourly or daily averages obtained from local meteorological stations. Two exceptions are a multicentre study conducted in China, which deployed outdoor temperature sensors in selected areas^39^ and a Canadian study^24^ that measured outdoor temperatures outside of the participants’ residences. Six studies^23 25 35 36 39^ examined the relationship between indoor and outdoor temperatures, identifying significant variations, particularly during colder periods.

Indoor and outdoor temperatures were positively correlated, with seasonal variations in correlation strength. In summer, Uejio *et al*^35^ found that indoor temperature increases by 0.16°C (95% CI 0.08 to 0.25) for every 1°C rise in outdoor temperature, with a 1-hour lag. The Masuda study^30^ also found high positive correlation between indoor and outdoor temperatures both in the morning and evening (Pearson correlation coefficient >0.852 and >0.799, respectively), although 95% CIs were not provided. Saeki *et al*^23^ reported that the correlation between indoor and outdoor temperature was weaker in winter (r=0.28) than spring/fall (r=0.84).

The indoor–outdoor temperature difference varied significantly by season and factors such as region, built environment, housing conditions and heating. Differences ranged from 0°C to 30°C across studies. During the 2018 heatwave in South Korea, outdoor temperatures averaged at 30.6°C (24.6–36.3°C), consistent with indoor temperatures (30.5°C, 22.9–38.3°C), but peak temperatures occurred indoors, with morning indoor temperatures (29.6°C) slightly exceeding outdoor (29.1°C, p<0.001).^25^ In contrast, in cold periods, Umishio *et al*^36^ reported indoor temperatures (morning: 14.5°C, evening: 17.8°C) were significantly higher than outdoor (morning: 3.6°C, evening: 5.0°C), with a 10–13°C difference. Saeki *et al*^23^ and Kubozono *et al*^28^ similarly found that indoor temperatures generally exceeded outdoor temperatures year-round. In winter, indoor temperatures were 9.7°C higher, reducing to 2.8°C in spring/fall.^23^ The differences between indoor and outdoor temperatures are influenced not only by seasonal variations but also by diurnal cycles and heating facilities. In New York,^35^ diurnal variations showed indoor temperatures (23.9°C) were generally lower than outdoor (25.6°C) during the day, but at night, indoor temperatures (22.9°C) exceeded outdoor (20.1°C) in 80.8% of buildings. In contrast, a multicentre study in China^39^ rural areas with traditional heating maintained higher indoor temperatures (17–22°C), while southern cities with limited heating infrastructure had indoor temperatures closely mirroring outdoor (8–18°C).

#### Adjustment for potential confounders

Statistical models in the included studies were adjusted for various confounders across demographic, lifestyle, health-related, socioeconomic and environmental domains.

Age (77.8%, 14/18) and sex (72.2%, 13/18) were the most frequently adjusted covariates, followed by body mass index (55.6%, 10/18). Lifestyle factors were less consistently considered, including smoking (50.0%, 9/18), alcohol consumption (44.4%, 8/18), physical activity (22.2%, 4/18) and dietary factors (33.3%, 6/18). Health-related variables, such as pre-existing medical conditions and medication use, were adjusted in 61.1% (11/18) of studies, while sleep variables were included in 27.8% (5/18). Socioeconomic indicators (eg, income or education) were considered in 22.2% (4/18) of studies. Environmental confounders were less frequently addressed: outdoor temperature was adjusted in 38.9% (7/18) of studies, followed by humidity (22.2%, 4/18) and air pollution (22.2%, 4/18), whereas solar radiation was rarely considered (5.6%, 1/18).

## Discussion

The relationship between indoor temperature and cardiovascular health, particularly in the context of climate change, is a growing area of environmental health research. With the increasing frequency and intensity of heatwaves, it is crucial to understand how prolonged exposure to high indoor temperature influences inhabitants’ health outcomes. While previous research has established links between environmental temperature and cardiovascular health, this scoping review provides a more specific overview of the evidence relating to indoor temperature exposure. The findings show that the current evidence base is strongly concentrated on BP and BP-related outcomes, with relatively limited evidence on autonomic responses, broader haemodynamic responses and other clinical cardiovascular events. In addition, substantial heterogeneity was identified in study populations, study designs, exposure assessment methods and adjustment for potential confounders. Taken together, these findings help clarify the current scope of evidence on indoor temperature and cardiovascular health and identify areas where important knowledge gaps remain.

### Health impacts of indoor temperature

The cardiovascular outcomes identified in this review can be broadly considered in terms of haemodynamic responses, acute autonomic responses and clinical cardiovascular morbidity and events.

#### Haemodynamic responses

One of the main findings from this scoping review is that there appears to be an inverse association between indoor temperature and BP. This association is particularly pronounced during colder periods, where lower indoor temperatures are linked to higher BP and increased hypertension risk.^29 37^ During high-temperature periods, several studies also reported a consistent inverse association between indoor temperature and BP.^25 28 31 32^

For example, elevated indoor temperatures during a heatwave were associated with reductions in DBP and increases in body temperature among older adults,^25^ suggesting that heat exposure can alter cardiovascular regulation in older adults. This decrease in BP may reflect vasodilation and sweating triggered by heat stress to dissipate body heat; however, these mechanisms can also reduce cardiac filling pressures and blood volume, potentially leading to oxygen supply-demand imbalance and increasing the risk of myocardial infarction or cardiovascular collapse in individuals with pre-existing cardiovascular conditions.^41^ Evidence from both experimental and observational studies further supports this relationship. Experimental studies have shown that hypertensive individuals may be more sensitive to short-term temperature changes^31^ and that prolonged exposure to elevated indoor temperatures can increase cardiovascular strain in older adults.^32^ Long-term observational evidence also indicates an inverse association between indoor temperature and BP, with possible seasonal variation in the strength of this relationship.^28^

BP variability was examined less frequently. Two studies reported associations between indoor temperature fluctuations and increased BP variability,^27 38^ suggesting that variation in indoor thermal conditions may also influence haemodynamic stability. Overall, however, the evidence on haemodynamic responses remains heavily concentrated on BP, with relatively limited investigation of other cardiovascular physiological measures.

#### Acute autonomic responses

Evidence on cardiac autonomic responses to indoor heat exposure was limited. Two studies specifically examined heart rate variability and other markers of cardiac autonomic function under elevated indoor temperature conditions.^32 33^ In a randomised crossover trial of older adults, exposure to 31°C and 36°C was associated with increased heart rate and reduced heart rate variability compared with exposure to 22°C.^32^ In a summer observational study of community-dwelling older adults, higher night-time bedroom temperatures were similarly associated with increased heart rate and reductions in several heart rate variability indices, with progressively greater changes at higher temperatures.^33^ These findings suggest that elevated indoor temperatures may influence cardiac autonomic regulation. However, given the small number of studies, the consistency, magnitude and clinical relevance of these responses remain uncertain.

#### Clinical cardiovascular morbidity and events

Evidence regarding cardiovascular morbidity and clinical events remains limited and inconsistent. A study in Taiwan reported a significant increase in cardiovascular emergency visits associated with high indoor temperatures (≥27°C) when cumulative degree hours exceeded 62 during the hot season.^34^ In contrast, studies conducted in New York found no clear association between indoor heat exposure and cardiovascular emergency outcomes.^35 40^ These differences may partly reflect variations in exposure assessment. The Taiwan study used predictive modelling and CDH to capture prolonged heat exposure, whereas the New York studies relied on direct measurements and the heat index to represent short-term exposure. Regional climatic conditions and housing environments may also contribute to these differences. Such methodological differences may limit comparability across studies, and the conflicting findings highlight the uncertainty regarding the impact of indoor temperature on cardiovascular outcomes during heat exposure.

Evidence from studies specifically focused on individuals with established cardiovascular disease other than hypertension was sparse. A panel study of patients with heart failure^24^ reported that higher indoor temperatures were associated with lower BP, reduced oxygen saturation, poorer self-rated health and worsening night-time shortness of breath. Beyond cardiovascular-specific outcomes, one study conducted during a heatwave reported several self-reported symptoms associated with indoor heat exposure, including dizziness, hyperhidrosis, fatigue, headache and dyspnoea.^25^ However, the study did not quantify the relationship between these symptoms and indoor temperature, limiting the ability to assess exposure-response relationships.

Evidence linking high indoor temperatures to cardiovascular mortality is lacking, with no relevant studies identified for this review. This aligns with the findings of another recent review.^42^ Several factors may contribute to this research gap. Mortality studies often demand robust study designs and longitudinal data, which are resource-intensive to obtain. The challenges in accurately identifying and attributing heat-related mortality to indoor temperature exposures make these studies particularly complex to design and interpret, especially when compared with other cardiovascular outcomes.

### Research gaps in study populations and design

Most reviewed studies focused on older adults, with half targeting those over 60 years and 27.8% on ages 50–60 years, reflecting a strong research emphasis on the vulnerability of older adults to indoor heat exposure. Older adults face higher chronic disease prevalence and reduced thermoregulation due to factors such as diminished sweating, cardiovascular changes, immune alterations and weaker heat stress perception.^43^ Those with cardiovascular conditions often show exaggerated BP changes before engaging in thermoregulatory behaviours.^44^ However, research on young and middle-aged adults remains limited. Further research across a broader age range is needed to determine whether the cardiovascular effects of indoor temperature differ by age and to inform age-specific interventions.

Most studies had relatively small sample sizes (16–401 participants), except one with over 260 000 participants. Studies with small sample sizes often came with a greater feasibility for personalised exposure assessment but such studies may be under-powered to detect moderate changes in temperature-related health outcomes. On the other hand, large-scale epidemiological studies, though able to capture diverse populations and extensive confounders, often rely on simplified or proxy measurements of indoor exposure.^34^ Recent development of personal temperature exposure model, by combining data from indoor temperature measurements, questionnaire and outdoor temperature measurements, has offered further research opportunities for large-scale epidemiological studies.^45^ More than half of the included studies used a cross-sectional design, which limits the ability to establish the temporal sequence between indoor temperature exposure and cardiovascular outcomes. Cross-sectional findings may also be susceptible to reverse causality and residual confounding from factors such as seasonality, medication use and recent physical activity. These associations should therefore be interpreted cautiously and should not be considered evidence of causality. Future longitudinal studies and experimental designs would help clarify temporality and strengthen causal inference.

Among the 18 included studies, only 5 conducted sex-stratified analyses.^25 26 30 34 36^ Overall, several studies suggested that women may exhibit greater cardiovascular sensitivity to indoor temperature variations, with stronger associations between temperature exposure and cardiovascular outcomes reported in female participants. For example, evidence indicates that women may experience increased cardiovascular risk at lower cumulative heat exposure thresholds and show larger BP responses to indoor temperature changes compared with men.^26 34 36^ Evidence was not entirely consistent, as two studies did not observe significant sex differences in cardiovascular responses to indoor temperature.^25 30^ In addition, few studies have investigated the physiological mechanisms underlying potential sex differences in heat-related cardiovascular responses, highlighting an important gap in the current literature.

Environmental factors were inconsistently adjusted across studies. Humidity, which affects thermoregulation by influencing sweat evaporation,^46^ was considered in only 22.2% (4/18) of studies, despite its critical role in the indoor temperature-health relationship. Meanwhile, another review^47^ suggested that humidity can influence temperature, and adjusting for it may remove its impact on both temperature and health outcomes, yielding a controlled direct effect of temperature rather than the total effect. It also highlighted the potential for multicollinearity between temperature and humidity, recommending the use of interaction terms to represent their combined effects or mass-based measures like absolute humidity for more accurate assessments. Both indoor and outdoor air pollution received even less attention in reviewed studies. While some reviews highlighted the common adjustment for outdoor air pollution in studies on outdoor heat exposure and cardiovascular outcomes,^7^ this scoping review found such adjustments for either outdoor or indoor air pollution were largely absent in studies on indoor temperature. Future studies should therefore consider more explicitly the potential roles of humidity and air pollution, while ensuring that covariate selection is guided by the hypothesised causal relationships between environmental exposures and cardiovascular outcomes.

### Methodological gaps in indoor temperature exposure assessments

Most studies adopted continuous monitoring, with monitors placed in the participant’s rooms throughout the measurement period, allowing high-resolution data and the capture of temporal temperature fluctuations. However, nearly one-third relied on a single measurement or measurements conducted over a short period, which may not adequately reflect variations in indoor temperature exposure. Evidence from studies included in this review, as well as previous reviews, indicates substantial diurnal variation in indoor temperature, including marked morning–evening differences.^25 38 48^ Study relying on single-time point measurements may therefore fail to capture these fluctuations.^37^ Even repeated measurements at limited time points (eg, two times per day recordings^25 36^), may miss critical exposure periods, including peak afternoon or early morning temperatures.

While continuous monitoring of indoor temperature provides more granular exposure data compared with one-off measurements, several practical limitations should be considered. Continuous monitoring devices may involve higher equipment costs and require additional maintenance and data-processing capacity, which may limit large-scale deployment. Additionally, participant compliance and privacy concerns may pose challenges for long-term monitoring, particularly when measurements are conducted within private indoor environments.^49^ To address these challenges, a hybrid approach combining questionnaires with short-term monitoring has been proposed.^39^ In this study, the questionnaire achieved a high recovery rate (over 98%) and collected data from 737 participants, while targeted monitoring was conducted in a smaller subsample of households. This approach enabled large-scale data collection while maintaining detailed environmental measurements, reducing monitoring costs and participant burden.

In most reviewed studies, sensors were placed predominantly in living rooms or bedrooms, aligning with areas where individuals spend substantial time indoors. However, indoor temperatures can vary across rooms. A study measuring summertime temperatures in 268 homes in Leicester, UK, found up to a 4°C difference between bedroom and living room temperatures.^50^ This spatial variation highlights that single-location measurements may not fully capture indoor heat exposure. Meanwhile, less attention has been given to other spaces, such as kitchens or bathrooms, despite activities like cooking or bathing potentially contributing to short-term heat exposure. Sensor placement height varied between 0.6 and 1.5 m, targeting the human breathing zone. However, temperature stratification within rooms affected by heating, cooling or ventilation may introduce measurement errors.^51^ Standardised guidelines have provided frameworks for consistent indoor temperature measurement.^52 53^ However, in practice, applying these standards in observational studies can be challenging due to diverse home environments and participant behaviours. Prescribed sensor placements may conflict with room layouts or participant preferences, and multisensor strategies, while methodologically robust, can be financially burdensome for large-scale studies.

Only 33.3% of the reviewed studies included individual behavioural data or housing characteristics in indoor temperature assessments. Although several studies collected time-activity information, reporting that participants spent most of their time indoors, these data were rarely incorporated into exposure models,^38 39^ representing a missed opportunity to improve exposure assessment. In contrast, Saeki *et al*^23^ have provided a compelling example of how integrating participants’ time-activity patterns into exposure assessment can enhance model performance. Nevertheless, the reliance on self-reported data may introduce recall bias or inaccuracies.

Housing characteristics have also been used to refine exposure assessment. Predictive models incorporating building features have been developed to estimate indoor temperatures and better characterise exposure.^34^ However, such models are often based on limited household samples and rely on estimated rather than directly measured temperatures, which may introduce uncertainty and limit their ability to capture regional variability.

Addressing these limitations, a pilot study by Ma *et al*^54^ suggested that using portable temperature loggers with armband sleeves provides an accurate and practical solution for real-world thermal monitoring. This approach improves data collection on complex thermal experiences and supports studying human responses.

### Future research directions

Research on the impacts from indoor high temperature on CVDs remains limited over the past decade. Insights from the WHO European Region’s Heat-Health Action Planning (HHAP) framework highlighted the importance of integrating comprehensive indoor temperature monitoring with health system preparedness to mitigate heat-related health risks. The HHAP emphasises the need for real-time surveillance, targeted support for vulnerable populations and long-term urban planning, providing a strategic perspective for addressing the multifaceted impacts of indoor overheating, particularly during heatwaves.^55^

Future research should prioritise under-represented groups, especially younger individuals, whose vulnerabilities to indoor thermal environments remain poorly understood. Combining large-scale cohort studies with smaller, targeted investigations can provide robust insights into how indoor temperatures affect cardiovascular health. Addressing confounding factors such as humidity and indoor air pollutants is essential from the perspectives of exposome. Additionally, exploring sex differences in physiological responses may reveal mechanisms influencing health outcomes. Technologies such as wearable sensors can enhance exposure assessment by providing more precise and comprehensive measurements, while integrating behavioural and housing factors can improve understanding of thermal stress. Beyond cardiovascular outcomes, heat-related symptoms such as heat exhaustion, cramps, strokes, anxiety, depression, dizziness and headaches require greater attention, particularly among individuals with cardiovascular conditions. Finally, qualitative research into heat perception, thermal comfort and adaptive behaviours can provide valuable insights, supporting the development of targeted interventions and informed policies.

### Limitations

This scoping review has several limitations. Only English-language publications were included, which may have led to the exclusion of relevant studies in other languages. While a comprehensive search strategy was employed, grey literature was not considered, potentially omitting unpublished or non-peer-reviewed studies that could contribute valuable insights. Consistent with the aims of a scoping review, no formal assessment of methodological quality or risk of bias was undertaken and the findings were not weighted according to study quality. In addition, more than half of the included studies were cross-sectional, limiting the ability to establish temporality and increasing the potential for reverse causality and residual confounding. Notably, the included studies exhibited substantial heterogeneity in terms of research design, population characteristics and temperature exposure assessment, which limited direct comparability across studies and precluded conclusions regarding the consistency and magnitude of observed associations.

## Conclusions

This scoping review shows that the current evidence on indoor temperature and cardiovascular health is concentrated predominantly on BP and BP-related outcomes, with several studies reporting an inverse association between indoor temperature and BP. Evidence on other haemodynamic measures, autonomic responses and clinical cardiovascular morbidity and events remains comparatively limited and, in some areas, inconsistent. Future research should prioritise longitudinal and experimental studies, improve the characterisation of personal indoor temperature exposure and examine a broader range of cardiovascular outcomes and vulnerable populations. Addressing these gaps will help strengthen the evidence needed to inform heat-health guidance and climate adaptation strategies for indoor environments.

## Supplementary material

10.1136/bmjopen-2026-123980 online supplemental file 1

10.1136/bmjopen-2026-123980 online supplemental file 2

10.1136/bmjopen-2026-123980 online supplemental file 3

## Data Availability

All data relevant to the study are included in the article or uploaded as supplementary information.

## References

[1] Yu S, Yan Z, Freychet N (2020). Trends in summer heatwaves in central Asia from 1917 to 2016: association with large‐scale atmospheric circulation patterns. Intl J Climatol.

[2] Yoon D, Cha D-H, Lee M-I (2020). Recent changes in heatwave characteristics over Korea. Clim Dyn.

[3] Trancoso R, Syktus J, Toombs N (2020). Heatwaves intensification in Australia: A consistent trajectory across past, present and future. Sci Total Environ.

[4] An N, Zuo Z (2021). Changing structures of summertime heatwaves over China during 1961–2017. Sci China Earth Sci.

[5] Chapman SC, Watkins NW, Stainforth DA (2019). Warming trends in summer heatwaves. Geophys Res Lett.

[6] World Meteorological Organization (2025). WMO confirms 2024 as warmest year on record at about 1.55°C above pre-industrial level. https://wmo.int/news/media-centre/wmo-confirms-2024-warmest-year-record-about-155degc-above-pre-industrial-level.

[7] Liu J, Varghese BM, Hansen A (2022). Heat exposure and cardiovascular health outcomes: a systematic review and meta-analysis. Lancet Planet Health.

[8] Ballester J, Quijal-Zamorano M, Méndez Turrubiates RF (2023). Heat-related mortality in Europe during the summer of 2022. Nat Med.

[9] Zhao Q, Guo Y, Ye T (2021). Global, regional, and national burden of mortality associated with non-optimal ambient temperatures from 2000 to 2019: a three-stage modelling study. Lancet Planet Health.

[10] Masselot P, Mistry M, Vanoli J (2023). Excess mortality attributed to heat and cold: a health impact assessment study in 854 cities in Europe. Lancet Planet Health.

[11] Rizmie D, de Preux L, Miraldo M (2022). Impact of extreme temperatures on emergency hospital admissions by age and socio-economic deprivation in England. Soc Sci Med.

[12] Coates L, van Leeuwen J, Browning S (2022). Heatwave fatalities in Australia, 2001–2018: an analysis of coronial records. Int J Disaster Risk Reduct.

[13] Duffield G, Bunn S (2023). Indoor air quality. Indoor Air.

[14] Mannan M, Al-Ghamdi SG (2021). Indoor Air Quality in Buildings: A Comprehensive Review on the Factors Influencing Air Pollution in Residential and Commercial Structure. Int J Environ Res Public Health.

[15] World Health Organization. Regional Office for Europe (2021). Heat and health in the WHO European Region: updated evidence for effective prevention.

[16] World Health Organization (2018). WHO housing and health guidelines: recommendation to promote healthy housing for a sustainable and equitable future.

[17] Erens B, Williams L, Exley J (2021). Public attitudes to, and behaviours taken during, hot weather by vulnerable groups: results from a national survey in England. BMC Public Health.

[18] Joe L, Hoshiko S, Dobraca D (2016). Mortality during a Large-Scale Heat Wave by Place, Demographic Group, Internal and External Causes of Death, and Building Climate Zone. Int J Environ Res Public Health.

[19] Wang Q, Li C, Guo Y (2017). Environmental ambient temperature and blood pressure in adults: A systematic review and meta-analysis. Sci Total Environ.

[20] Tham S, Thompson R, Landeg O (2020). Indoor temperature and health: a global systematic review. Public Health.

[21] Peters MDJ, Marnie C, Tricco AC (2020). Updated methodological guidance for the conduct of scoping reviews. *JBI Evid Synth*.

[22] Tricco AC, Lillie E, Zarin W (2018). PRISMA Extension for Scoping Reviews (PRISMA-ScR): Checklist and Explanation. Ann Intern Med.

[23] Saeki K, Obayashi K, Iwamoto J (2014). The relationship between indoor, outdoor and ambient temperatures and morning BP surges from inter-seasonally repeated measurements. J Hum Hypertens.

[24] Goldberg MS, Wheeler AJ, Burnett RT (2015). Physiological and perceived health effects from daily changes in air pollution and weather among persons with heart failure: a panel study. J Expo Sci Environ Epidemiol.

[25] Kim S, Kim S-Y, Oh J (2020). Effects of the 2018 heat wave on health in the elderly: implications for adaptation strategies to climate change. Environ Anal Health Toxicol.

[26] Ni Y, Miao Q, Zheng R (2020). Individual sensitivity of cold pressor, environmental meteorological factors associated with blood pressure and its fluctuation. Int J Biometeorol.

[27] Chen T, Ge J, Luo X (2023). Effects of indoor temperature and its fluctuation on blood pressure and its variability. Int J Biometeorol.

[28] Kubozono T, Akasaki Y, Kawasoe S (2021). The relationship between home blood pressure measurement and room temperature in a Japanese general population. Hypertens Res.

[29] Li Y, Gu Y, Li Z (2023). Study on the challenge and influence of the built thermal environment on elderly health in rural areas: evidence from Shandong, China. Build Simul.

[30] Kinuta M, Hisamatsu T, Fukuda M (2023). Associations of indoor and outdoor temperatures and their difference with home blood pressure: The Masuda Study. Hypertens Res.

[31] Chen X, Tu P, Sun X-L (2021). The Impact on Blood Pressure of a Short-Term Change in Indoor Temperature. Int J Gen Med.

[32] Meade RD, Akerman AP, Notley SR (2024). Effects of Daylong Exposure to Indoor Overheating on Thermal and Cardiovascular Strain in Older Adults: A Randomized Crossover Trial. Environ Health Perspect.

[33] O’Connor FK, Bach AJE, Forbes C (2025). Effect of nighttime bedroom temperature on heart rate variability in older adults: an observational study. BMC Med.

[34] Jung C-C, Hsia Y-F, Hsu N-Y (2020). Cumulative effect of indoor temperature on cardiovascular disease-related emergency department visits among older adults in Taiwan. Sci Total Environ.

[35] Uejio CK, Tamerius JD, Vredenburg J (2016). Summer indoor heat exposure and respiratory and cardiovascular distress calls in New York City, NY, U.S. Indoor Air.

[36] Umishio W, Ikaga T, Kario K (2019). Cross-Sectional Analysis of the Relationship Between Home Blood Pressure and Indoor Temperature in Winter: A Nationwide Smart Wellness Housing Survey in Japan. Hypertension.

[37] Zhao H, Jivraj S, Moody A (2019). “My blood pressure is low today, do you have the heating on?” The association between indoor temperature and blood pressure. J Hypertens.

[38] Umishio W, Ikaga T, Kario K (2021). Impact of indoor temperature instability on diurnal and day-by-day variability of home blood pressure in winter: a nationwide Smart Wellness Housing survey in Japan. Hypertens Res.

[39] Song B, Yoshino H, Goto T (2022). Indoor thermal environment during heating season and the health of elderly in China. Sci Technol Built Environ.

[40] Gonsoroski E, Tamerius JD, Asaeda G (2025). Respiratory and Cardiovascular Medical Emergency Calls Related to Indoor Heat Exposure through a Case-Control Study in New York City. J Urban Health.

[41] Ebi KL, Capon A, Berry P (2021). Hot weather and heat extremes: health risks. Lancet.

[42] Singh N, Areal AT, Breitner S (2024). Heat and Cardiovascular Mortality: An Epidemiological Perspective. Circ Res.

[43] Millyard A, Layden JD, Pyne DB (2020). Impairments to Thermoregulation in the Elderly During Heat Exposure Events. Gerontol Geriatr Med.

[44] Schlader ZJ, Coleman GL, Sackett JR (2018). Behavioral thermoregulation in older adults with cardiovascular co-morbidities. Temperature (Austin).

[45] Xia X, Chan KH, Niu Y (2024). Modelling personal temperature exposure using household and outdoor temperature and questionnaire data: Implications for epidemiological studies. Environ Int.

[46] Sobolewski A, Młynarczyk M, Konarska M (2021). The influence of air humidity on human heat stress in a hot environment. Int J Occup Saf Ergon.

[47] Sharma R, Schinasi LH, Lee BK (2024). Air Pollution and Temperature in Seizures and Epilepsy: A Scoping Review of Epidemiological Studies. Curr Environ Health Rep.

[48] Salthammer T, Morrison GC (2022). Temperature and indoor environments. Indoor Air.

[49] Coulby G, Clear AK, Jones O (2021). Low-cost, multimodal environmental monitoring based on the Internet of Things. Build Environ.

[50] Lomas KJ, Kane T (2013). Summertime temperatures and thermal comfort in UK homes. Building Res Inform.

[51] Wang LL, Zhang X, Qi D (2019). Indoor thermal stratification and its statistical distribution. Indoor Air.

[52] Ramspeck CB (2004). Thermal environmental conditions for human occupancy.

[53] International Organization for Standardization (1998). Ergonomics of the thermal environment—instruments for measuring physical quantities.

[54] Ma R, Fu Y, Deng M (2020). Measurement of Personal Experienced Temperature Variations in Rural Households Using Wearable Monitors: A Pilot Study. Int J Environ Res Public Health.

[55] Martinez GS, Kendrovski V, Salazar MA (2022). Heat-health action planning in the WHO European Region: Status and policy implications. Environ Res.

