# Supplementary material for: Impacts of indoor high temperature on cardiovascular health among community-dwelling adults: a scoping review
Source: BMJ Open. 2026 Nov 6;16(11):e123980. doi: 10.1136/bmjopen-2026-123980 (PMC13756353; doi:10.1136/bmjopen-2026-123980)
Supplement: Supplementary file 1 [file bmjopen-2026-123980.reviewer_comments.pdf]

# PEER REVIEW HISTORY

BMJ Open publishes all reviews undertaken for accepted manuscripts. Reviewers are asked to complete a checklist review form and are provided with free text boxes to elaborate on their assessment. These free text comments are reproduced below.

## ARTICLE DETAILS

### Title (Provisional)

Impacts of indoor high temperature on cardiovascular health among community-dwelling adults: A scoping review

### Authors

Liao, Jianying; Ray-Bennett, Nibedita S.; Vande Hey, Joshua; Yang, Liu; Cai, Samuel

---

## VERSION 1 - REVIEW

---

| | |
|---|---|
| **Reviewer** | **1** |
| **Name** | **Gökdemir, Özden** |
| **Affiliation** | **Izmir University of Economics, Family Medicine** |
| **Date** | **19-Aug-2026** |
| **COI** | **NA** |

---

Dear Author/s,

Thank you very much for this study. Would you like to improve some details?

1)Although the goal is to "map and summarise," by definition, a scoping review does not usually evaluate the included studies' quality or bias risk. Nonetheless, the narrative synthesis of the review gives equal weight to data from cohort and experimental studies (each n=2) and cross-sectional designs (n=10, 55.6%). This is a crucial epidemiological problem. Cross-sectional studies are unable to establish temporal sequences and are very vulnerable to reverse causality. For example, seasonality, medication adherence, or recent physical activity may obfuscate an observed relationship between interior temperature and blood pressure on a single day.

2) The other structure could be

a) Acute autonomic responses (e.g., heart rate variability, which is only mentioned briefly).

b)Haemodynamic strain (e.g., cardiac output, stroke volume).

c) Clinical events (e.g., arrhythmias, myocardial infarction, stroke).

Maybe a meaningful taxonomy of cardiovascular impacts, instead of passively reflecting the literature's bias, could be more relevant

| | |
|---|---|
| **Reviewer** | **2** |
| **Name** | **Sever, Peter** |
| **Affiliation** | **Imperial College London, International Centre for Circulatory Health** |
| **Date** | **06-Sep-2026** |
| **COI** | **NA** |

This is an Interesting and timely review of the potential impact of variation in indoor temperature and cardiovascular morbidities. It is an important topic in the context of the anticipated changes due to global warming.

This is a scoping review and reports on a wide heterogeneity of individual studies that lack any consistency in methodology with respect to measurements of temperature and recordings of health outcomes. Amongst these widely varying studies there seems to be clear evidence that higher temperatures are associated with lowering of blood pressure levels and that this is more evident in hypertensive patients. These findings are commensurate with many earlier studies reporting that lower environmental temperature is associated with higher blood pressure levels and increased cardiovascular risk. The second finding is that cardiovascular emergencies are reported more commonly in association with high indoor temperatures and that this is particularly evident in older people in whom it is presumed cardiovascular homeostasis is compromised compared with younger subjects

The review also identifies a variety of other factors influenced by temperature including blood pressure variability, which has been shown to be an independent cardiovascular risk factor, which increases with increasing environmental temperature and respiratory distress.

I am not sure that we have learned anything new from this analysis. It simply serves to remind us that cardiovascular morbidity and mortality which are globally the predominant causes of death and disability will inevitably be influenced by global warming through direct effects of environmental temperature on the cardiovascular system particularly in older subjects and those with pre-existing cardiovascular disease.

## VERSION 1 - AUTHOR RESPONSE

### Reviewer 1

We thank the reviewer for these helpful comments, which prompted us to strengthen the interpretation of the evidence and improve the organisation of the Discussion.

## Comment 1

*"Although the goal is to 'map and summarise,' by definition, a scoping review does not usually evaluate the included studies' quality or bias risk. Nonetheless, the narrative synthesis of the review gives equal weight to data from cohort and experimental studies (each n=2) and cross-sectional designs (n=10, 55.6%). This is a crucial epidemiological problem. Cross-sectional studies are unable to establish temporal sequences and are very vulnerable to reverse causality. For example, seasonality, medication adherence, or recent physical activity may obfuscate an observed relationship between interior temperature and blood pressure on a single day."*

**Response:**

We agree with the reviewer that the predominance of cross-sectional studies is important when interpreting the findings. As this is a scoping review, we did not introduce a formal risk-of-bias assessment or assign quantitative weights to individual studies. Instead, we have revised the Discussion to make the implications of study design more explicit and to avoid interpreting cross-sectional associations as evidence of causality.

Specifically, we now state that more than half of the included studies used cross-sectional designs, which limits the ability to establish the temporal sequence between indoor temperature exposure and cardiovascular outcomes. We have also explicitly acknowledged the potential for reverse causality and residual confounding from factors including seasonality, medication use and recent physical activity. We further emphasise the need for longitudinal and experimental studies to clarify temporality and strengthen causal inference.

The Limitations section has also been revised to clarify that no formal methodological quality or risk-of-bias assessment was undertaken and that findings were not weighted according to study quality.

Changes made:

Discussion, Research gaps in study populations and design:

"More than half of the included studies used a cross-sectional design, which limits the ability to establish the temporal sequence between indoor temperature exposure and cardiovascular outcomes. Cross-sectional findings may also be susceptible to reverse causality and residual confounding from factors such as seasonality, medication use and recent physical activity. These associations should therefore be interpreted cautiously and should not be considered evidence of causality. Future longitudinal studies and experimental designs would help clarify temporality and strengthen causal inference."

Limitations:

"Consistent with the aims of a scoping review, no formal assessment of methodological quality or risk of bias was undertaken, and the findings were not weighted according to study quality. In addition, more than half of the included studies were cross-sectional,

limiting the ability to establish temporality and increasing the potential for reverse causality and residual confounding."

## Comment 2

*"The other structure could be*

*a) Acute autonomic responses (e.g., heart rate variability, which is only mentioned briefly).*

*b) Haemodynamic strain (e.g., cardiac output, stroke volume).*

*c) Clinical events (e.g., arrhythmias, myocardial infarction, stroke).*

*Maybe a meaningful taxonomy of cardiovascular impacts, instead of passively reflecting the literature's bias, could be more relevant."*

**Response:**

Thank you for this helpful suggestion. We agreed that organising the cardiovascular findings according to physiological and clinical domains would provide a more informative synthesis. We have therefore substantially reorganised the health impacts of indoor temperature section of the Discussion into three domains:

1. Haemodynamic responses

2. Acute autonomic responses

3. Clinical cardiovascular morbidity and events

The haemodynamic section now synthesises evidence on blood pressure and blood pressure variability and explicitly notes that the existing evidence is strongly concentrated on blood pressure, with relatively limited investigation of other cardiovascular physiological measures.

The autonomic section has been expanded. We now discuss the two studies that specifically investigated heart rate variability and other markers of cardiac autonomic function under elevated indoor temperature conditions, including evidence from a randomised crossover trial and a summer observational study.

The clinical cardiovascular section separately considers cardiovascular emergency visits/calls, outcomes among individuals with established cardiovascular disease, broader heat-related symptoms and the absence of evidence on cardiovascular mortality.

Changes made:

The revised section now begins with:

"The cardiovascular outcomes identified in this review can be broadly considered in terms of haemodynamic responses, acute autonomic responses, and clinical cardiovascular morbidity and events."

Under Haemodynamic responses, we consolidated the evidence on blood pressure and blood pressure variability and added a clearer synthesis highlighting that the existing evidence is concentrated predominantly on blood pressure-related outcomes.

Under Acute autonomic responses, we expanded the previous brief discussion of cardiac autonomic function into a separate subsection, synthesising the available evidence on heart rate and heart rate variability.

Under Clinical cardiovascular morbidity and events, we reorganised the evidence on cardiovascular emergency outcomes, findings among individuals with established cardiovascular disease, heat-related symptoms and cardiovascular mortality.

Together, these revisions provide a clearer and more clinically meaningful synthesis of the cardiovascular outcomes and make the current gaps across different outcome domains more explicit.

## Reviewer 2

We thank the reviewer for recognising the timeliness and importance of this topic and for highlighting the heterogeneity of the existing evidence.

## Comment

*"This is an Interesting and timely review of the potential impact of variation in indoor temperature and cardiovascular morbidities. It is an important topic in the context of the anticipated changes due to global warming.*

*This is a scoping review and reports on a wide heterogeneity of individual studies that lack any consistency in methodology with respect to measurements of temperature and recordings of health outcomes. Amongst these widely varying studies there seems to be clear evidence that higher temperatures are associated with lowering of blood pressure levels and that this is more evident in hypertensive patients. These findings are commensurate with many earlier studies reporting that lower environmental temperature is associated with higher blood pressure levels and increased cardiovascular risk. The second finding is that cardiovascular emergencies are reported more commonly in association with high indoor temperatures and that this is particularly evident in older people in whom it is presumed cardiovascular homeostasis is compromised compared with younger subjects.*

*The review also identifies a variety of other factors influenced by temperature including blood pressure variability, which has been shown to be an independent cardiovascular risk factor, which increases with increasing environmental temperature and respiratory distress.*

*I am not sure that we have learned anything new from this analysis. It simply serves to remind us that cardiovascular morbidity and mortality which are globally the predominant causes of death and disability will inevitably be influenced by global warming through direct effects of environmental temperature on the cardiovascular system particularly in older subjects and those with pre-existing cardiovascular disease."*

**Response:**

We thank the reviewer for this thoughtful summary of our findings and for highlighting the importance of this topic in the context of climate change. We agree that the broader relationship between environmental temperature and cardiovascular health, particularly the inverse relationship between temperature and blood pressure, has been established previously.

The specific contribution of this scoping review is to map the evidence relating specifically to indoor temperature exposure and to characterise how that evidence is distributed across cardiovascular outcomes, study designs and exposure assessment approaches. Our review shows that the current indoor-temperature evidence base is concentrated predominantly on blood pressure and blood pressure-related outcomes, while evidence on other haemodynamic measures, autonomic responses and clinical cardiovascular morbidity and events remains limited and heterogeneous. We also identified substantial methodological heterogeneity in study populations, study designs, exposure assessment methods and adjustment for potential confounders.

We have revised the Discussion and Conclusion to make these overall findings and their implications clearer. In particular, we now emphasise the specific gaps in the indoor-temperature evidence base and the need for stronger longitudinal and experimental studies, improved characterisation of indoor temperature exposure, and investigation of a broader range of cardiovascular outcomes. Addressing these gaps may help inform future heat-health guidance and climate adaptation strategies for indoor environments.

Change made:

We made several revisions to clarify the overall findings and contribution of the review.

First, we revised the Abstract Results to more clearly show the distribution of the available evidence across cardiovascular outcome domains, highlighting that blood pressure and blood pressure-related outcomes predominate, while substantially fewer studies examined other haemodynamic measures, cardiac autonomic responses or clinical cardiovascular morbidity and events.

Second, we revised the opening paragraph of the Discussion to clarify the specific contribution of the review and to distinguish the evidence relating to indoor temperature exposure from the broader literature on environmental temperature and cardiovascular health. We now explicitly highlight the concentration of the evidence on blood pressure-related outcomes, as well as the heterogeneity in study populations, study designs, exposure assessment methods and adjustment for potential confounders.

Third, we reorganised the health impacts of indoor temperature section into three cardiovascular outcome domains: Haemodynamic responses, Acute autonomic responses, and Clinical cardiovascular morbidity and events. This restructuring provides a clearer

synthesis of the available evidence and makes the gaps across cardiovascular outcome domains more explicit.

Finally, we revised the Conclusion to emphasise that the current evidence base is concentrated predominantly on blood pressure and blood pressure-related outcomes, while evidence on other haemodynamic measures, autonomic responses and clinical cardiovascular morbidity and events remains comparatively limited and, in some areas, inconsistent. We also emphasise priorities for future research, including stronger longitudinal and experimental studies, improved characterisation of personal indoor temperature exposure, and investigation of a broader range of cardiovascular outcomes and vulnerable populations, with the aim of informing future heat-health guidance and climate adaptation strategies for indoor environments.

We thank the Editor and both reviewers again for their constructive comments. We hope that the revisions have addressed the points raised and improved the clarity and usefulness of the manuscript.
