# Supplementary material for: Impacts of indoor high temperature on cardiovascular health among community-dwelling adults: a scoping review
Source: BMJ Open. 2026 Nov 6;16(11):e123980. doi: 10.1136/bmjopen-2026-123980 (PMC13756353; doi:10.1136/bmjopen-2026-123980)
Supplement: Supplementary file 2 [file bmjopen-16-11-s001.docx]

**Supplementary file 1**

Supplementary Material A. Database search strategies

Keyword and query

keyword

To ensure a thorough search, we used a combination of Medical Subject Headings (MeSH) and free-text terms. The primary search terms included:

- “Indoor high temperature” OR “Indoor heat” OR “Indoor heat exposure”
- “Cardiovascular diseases” [MeSH] OR “CVDs” OR “heart disease” OR “Hypertension” [MeSH] OR “High blood pressure”

Query

These terms were combined using Boolean operators (AND, OR) to refine the search based on different database

PubMed:

(("indoor heat") OR ("personal heat exposure") OR ("indoor temperature")) AND (("cardiovascular diseases"[MeSH]) OR ("CVDs") OR ("heart disease") OR ("hypertension"[MeSH]) OR ("high blood pressure") OR (hypertensive))

Medline:

1	indoor high temperature.mp.

2	indoor heat.mp.

3	personal heat exposure.mp.

4	Indoor thermal environment.mp.

5	hypertension.mp.

6	high blood pressure.mp.

7	Cardiovascular disease.mp.

8	CVDs.mp.

9	hypertensive.mp.

10	1 or 2 or 3 or 4

11	5 or 6 or 7 or 8 or 9

12	10 and 11

Web of science:

(ALL=(indoor high temperature) OR ALL=(indoor heat)) AND (ALL=(hypertension) OR ALL=(cardiovascular disease))

Supplementary Material B. Full list of the included studies

1. Saeki K, Obayashi K, Iwamoto J, Tone N, Okamoto N, Tomioka K, et al. The relationship between indoor, outdoor and ambient temperatures and morning BP surges from inter-seasonally repeated measurements. Journal of Human Hypertension. 2014 2014/08/01;28(8):482-8.

2. Goldberg MS, Wheeler AJ, Burnett RT, Mayo NE, Valois M-F, Brophy JM, et al. Physiological and perceived health effects from daily changes in air pollution and weather among persons with heart failure: A panel study. Journal of Exposure Science & Environmental Epidemiology. 2015 2015/03/01;25(2):187-99.

3. Uejio CK, Tamerius JD, Vredenburg J, Asaeda G, Isaacs DA, Braun J, et al. Summer indoor heat exposure and respiratory and cardiovascular distress calls in New York City, NY, U.S. Indoor Air. 2016 Aug;26(4):594-604. PubMed PMID: 26086869. PMCID: PMC4786471. Epub 20150708. eng.

4. Umishio W, Ikaga T, Kario K, Fujino Y, Hoshi T, Ando S, et al. Cross-Sectional Analysis of the Relationship Between Home Blood Pressure and Indoor Temperature in Winter: A Nationwide Smart Wellness Housing Survey in Japan. Hypertension. 2019 Oct;74(4):756-66. PubMed PMID: 31446802. Epub 20190826. eng.

5. Zhao H, Jivraj S, Moody A. ‘My blood pressure is low today, do you have the heating on?’ The association between indoor temperature and blood pressure. Journal of Hypertension. 2019;37(3).

6.Jung CC, Hsia YF, Hsu NY, Wang YC, Su HJ. Cumulative effect of indoor temperature on cardiovascular disease-related emergency department visits among older adults in Taiwan. Sci Total Environ. 2020 Aug 20;731:138958. PubMed PMID: 32408209. Epub 20200428. eng.

7. Kim S, Kim SY, Oh J, Chae Y, Park J, Kim D, et al. Effects of the 2018 heat wave on health in the elderly: implications for adaptation strategies to climate change. Environ Anal Health Toxicol. 2020 Dec;35(4):e2020024-0. PubMed PMID: 33434424. PMCID: PMC7829408. Epub 20201221. eng.

8. Ni Y, Miao Q, Zheng R, Miao Y, Zhang X, Zhu Y. Individual sensitivity of cold pressor, environmental meteorological factors associated with blood pressure and its fluctuation. Int J Biometeorol. 2020 Sep;64(9):1509-17. PubMed PMID: 32415619. Epub 20200515. eng.

9. Chen X, Tu P, Sun XL, Hu TY, Wan J, Hu YW, et al. The Impact on Blood Pressure of a Short-Term Change in Indoor Temperature. Int J Gen Med. 2021;14:1507-11. PubMed PMID: 33911895. PMCID: PMC8075305. Epub 20210422. eng.

10. Kubozono T, Akasaki Y, Kawasoe S, Ojima S, Kawabata T, Makizako H, et al. The relationship between home blood pressure measurement and room temperature in a Japanese general population. Hypertension Research. 2021 2021/04/01;44(4):454-63.

11. Umishio W, Ikaga T, Kario K, Fujino Y, Suzuki M, Ando S, et al. Impact of indoor temperature instability on diurnal and day-by-day variability of home blood pressure in winter: a nationwide Smart Wellness Housing survey in Japan. Hypertension Research. 2021 2021/11/01;44(11):1406-16.

12. Song B, Yoshino H, Goto T, Zhang H, Guan J, Hasegawa K, et al. Indoor thermal environment during heating season and the health of elderly in China. Science and Technology for the Built Environment. 2022 2022/08/09;28(7):843-63.

13. Li Y, Gu Y, Li Z, Zhang X, Gao W, Xiao F. Study on the challenge and influence of the built thermal environment on elderly health in rural areas: Evidence from Shandong, China. Building Simulation. 2023 2023/08/01;16(8):1345-60.

14. Kinuta M, Hisamatsu T, Fukuda M, Taniguchi K, Komukai S, Nakahata N, et al. Associations of indoor and outdoor temperatures and their difference with home blood pressure: The Masuda Study. Hypertension Research. 2023 2023/01/01;46(1):200-7.

15. Chen T, Ge J, Luo X. Effects of indoor temperature and its fluctuation on blood pressure and its variability. Int J Biometeorol. 2023 Aug;67(8):1279-90. PubMed PMID: 37410169. Epub 20230706. eng.

16. Meade RD, Akerman AP, Notley SR, Kirby NV, Sigal RJ, Kenny GP. Effects of Daylong Exposure to Indoor Overheating on Thermal and Cardiovascular Strain in Older Adults: A Randomized Crossover Trial. Environ Health Perspect. 2024 Feb;132(2):27003. PubMed PMID: 38329752. PMCID: PMC10852046. Epub 20240208. eng.

17. Gonsoroski E, Tamerius JD, Asaeda G, Isaacs DA, Braun J, Remigio R, et al. Respiratory and Cardiovascular Medical Emergency Calls Related to Indoor Heat Exposure through a Case-Control Study in New York City. J Urban Health. 2025 Feb;102(1):177-88. PubMed PMID: 39870982. PMCID: PMC11865379. eng.

18. O'Connor FK, Bach AJE, Forbes C, Rutherford S, Binnewies S, Sabapathy S, et al. Effect of nighttime bedroom temperature on heart rate variability in older adults: an observational study. BMC Med. 2025 Dec 29;23(1):703. PubMed PMID: 41462246. PMCID: PMC12751657. Epub 20251229. eng.
