# Supplementary material for: Impacts of indoor high temperature on cardiovascular health among community-dwelling adults: a scoping review
Source: BMJ Open. 2026 Nov 6;16(11):e123980. doi: 10.1136/bmjopen-2026-123980 (PMC13756353; doi:10.1136/bmjopen-2026-123980)
Supplement: Supplementary file 3 [file bmjopen-16-11-s002.docx]

**Supplementary File 2**

Table S1. Full characteristics of included studies

| Study | Design | Region | Population | Population characters (age & sex) (n/%) | Health outcomes | Confounder | Key findings |
| --- | --- | --- | --- | --- | --- | --- | --- |
| Saeki et al. (2014) | Cross-sectional | Nara, Japan | Elderly individuals living at home, aged 60 years or older | 192; 96 (50.0%) were men; mean age: 69.9±6.3 yrs | Morning blood pressure surge (MBPS), including sleep-trough MBPS and pre waking MBPS | Physical activity, age, sex, BMI, smoking and drinking habits, medication use (antihypertensives, antidiabetics, lipid-lowering agents), socioeconomic status (income, education), sleep profiles. | A 1°C decrease in ambient temperature was associated with a 0.44 mmHg (95% CI: 0.23–0.65) increase in sleep-trough MBPS and a 0.52 mmHg (95% CI: 0.31–0.72) increase in pre waking MBPS, independent of potential confounders. |
| Goldberg et al. (2015) | Panel | Montreal, Canada | Individuals with heart failure | 55; 50 (90.9%) were men. 15 (27.3%) aged 35–55 yrs, 12 (21.8%) aged 56–61 yrs, 14 (25.5%) aged 62–66 yrs, and 14 (25.5%) aged 67 yrs and above.  Median age: 62 yrs | Oxygen saturation, pulse rate, SBP, DBP, self-rated health, shortness of breath at night. | Age, sex, weight, body temperature, liquid consumption, salt intake, illness, sleeping problems, month of the year, outdoor temperature, air pollution, humidity. | In individuals with heart failure, oxygen saturation decreased with increasing outdoor temperature (-0.34%, 95% CI: -0.54, -0.13) and indoor temperature (-0.14%, 95% CI: -0.25, -0.03); SBP decreased with increasing outdoor temperature (-0.91 mmHg, 95% CI: -1.98, 0.75) and indoor temperature (-0.99 mmHg, 95% CI: -2.01, 0.03); self-rated health deteriorated with indoor temperature (-2.13, 95% CI: -3.11, -1.15) and outdoor temperature (-1.15, 95% CI: -3.00, -0.70); and shortness of breath at night worsened with increasing indoor temperature (-2.32, 95% CI: -3.67, -0.98). |
| Uejio et al. (2016) | Case-control | New York City, USA | Individuals receiving emergency medical care during summer | 1,100 (764 after exclusion); 427 (56%) were men; The median age: 52 yrs | Cardiovascular distress calls | Age, sex, pre-existing medical conditions, income, racial composition, vacancy rates, living alone percentages, outdoor temperature, humidity, wind speed, solar radiation, air pollution (Ozone, PM2.5), time of day. | Indoor temperatures increased by 0.16°C for every 1°C rise in outdoor temperature (P < 0.001), older patients experienced 0.09°C warmer indoor environments per decade (P = 0.014), and while indoor heat indices ≥26°C increased the odds of respiratory distress by 63% (OR: 1.63, 95% CI: 0.98–2.68, P = 0.056), no significant association was found with cardiovascular distress (OR: 0.85, 95% CI: 0.55–1.31, P = 0.472). |
| Umishio et al. (2019) | Cross-sectional | Japan | Individuals aged 20 years or older from households intending to perform insulation retrofitting | 3,775 (2,900 After exclusion); 1, 658 (47.2%) were men; mean age: 57±13 yrs | Home blood pressure (morning and evening systolic and diastolic blood pressure).  Heart rate. | Age, sex, BMI, smoking, alcohol consumption, salt intake, vegetable consumption, antihypertensive drug use, outdoor temperature, quality of sleep, sleep duration, temperature disparity between rooms. | Participants aged 57 years (mean age in this survey) had an average increase in morning/evening SBP of 8.2 (−0.86 to −0.77)/6.5 (−0.70 to −0.59) mm Hg per 10°C decrease in indoor ambient temperature; The relationship between SBP and indoor temperature was cubic, with sensitivity varying across temperature ranges; A 10°C decrease in indoor temperature led to varying increases in morning SBP: 3.8mmHg in 30-year-old men, 7.6mmHg in 60-year-old men, and 10.2mmHg in 80-year-old men. For women, the increases were 5.3mmHg, 9.1mmHg, and 11.6mmHg, respectively. |
| Zhao et al. (2019) | Cross-sectional | England | Community-dwelling adults aged 16 and over | 4,659; 2,056 (44.1%) were men; mean age: 47.6 ± 19.08 yrs | SBP, DBP | Age, sex, marital status, ethnicity, highest educational qualification, equivalised household income, area deprivation (IMD), BMI, mental health (GHQ-12 score), type 2 diabetes diagnosis, total cholesterol level, urine sodium excretion, alcohol consumption, smoking status, physical activity levels, outdoor temperature. | A 1°C decrease in indoor temperature was associated with a 0.48 (95% CI −0.72 to −0.25) mmHg increase in SBP and a 0.45 (95% CI −0.63 to −0.27) mmHg increase in DBP. The relationship was stronger in individuals who engaged in less physical activity. |
| Jung et al. (2020) | Cross-sectional | Taiwan | Aged 65 years and above. | 260,465; 131,537(50.5%) were men; Categorised into three age groups: 65–74 years, 75–84 years, and ≥85 years. | Emergency department visits related to CVDs. | Air pollution (PM2.5, O3, NOx), day of the week, holidays, time trends, and seasonality. | During the warm season, the risk on CVD emergencies increased significantly when CDH exceeded 62 hours at 27°C (RR=1.18, 95% CI: 1.01 to 1.39) and continued to rise as the temperature increased. Similarly, during the cold season, a significant risk was observed when CDH exceeded 47 hours at 23°C (RR=1.11, 95% CI: 1.00 to 1.23). |
| Kim et al. (2020) | Cross-sectional | Gijang (Busan), Imsil (Jeollabuk-do), Gwangyang (Jeollanam-do), and Namhae (Gyeongsangnam-do), South Korea | Elderly individuals aged 65 years and older | 104; 29 (27.9%) were men; mean age: 79.6 yrs. | Body temperature, SBP, DBP, sleep hours, heat related symptoms. | Age, sex, smoking status, medication use, and indoor humidity. | Over half (58.7%) of participants reported sleep disturbances during the heat wave, highlighting the vulnerability of the elderly to high indoor temperatures.  DBP decreased by 0.44 (95% CI: -0.84 to -0.04) mmHg for each 1°C increase in indoor temperature among elderly individuals with hypertension |
| Ni et al. (2020) | Cohort | Hangzhou city, China | Healthy individuals aged 18–60 years | 48; 17 (35.4%) were men; The mean age: 36.3 ± 5.8 yrs | SBP, DBP; Maximum difference (Maxdiff) and the standard deviation (SD) of SBP and DBP to quantify blood pressure fluctuations. | Age, sex, BMI, waist circumference (WC), waist-to-hip ratio (WHR), smoking, alcohol consumption, physical activity, baseline blood pressure, outdoor temperature, barometric pressure, humidity, time of measurement. | Indoor temperature was independently associated with blood pressure, with a decrease of 0.075 mmHg (95% CI: -0.145, -0.004, P = 0.038) in SBP and 0.117 mmHg (95% CI: -0.239, -0.133, P < 0.001) in DBP per 1°C increase. Humidity was independently associated with blood pressure, with a decrease of 0.042 mmHg (95% CI: -0.068, -0.016, P = 0.001) in SBP and 0.036 mmHg (95% CI: -0.058, -0.015, P = 0.001) in DBP per 1% increase |
| Chen et al. (2021) | Experimental | Nanchang, China | 55 individuals with hypertension and 57 without hypertension | 112; 56 (50.0%) were men; The mean age: Hypertension group: 57.7 ± 12.6 yrs, non-hypertension group: 23.1 ± 2.4 yrs | SBP, DBP; Observed BP changes were recorded as delta BP, reflecting the variation induced by temperature differences. | Baseline BP, age, sex distribution, and presence of hypertension were accounted for in analysis. Participants were instructed to abstain from caffeine, alcohol, and strenuous physical activity before testing. | A 10°C decrease in room temperature caused an SBP increase of 4.1 mmHg (95% CI: 3.528–6.431) and a DBP increase of 3.6 mmHg (95% CI: 1.687–5.551), while a 10°C increase caused an SBP decrease of 4.0 mmHg (95% CI: 1.444–6.592) and a non-significant DBP decrease of 1.3 mmHg (95% CI: -0.874–3.370).  Hypertensive participants exhibited a significantly larger SBP response |
| Kubozono et al. (2021) | Cross-sectional | Tarumizu City, Japan | Community-living adults aged 40 years or older | 401 (395 after exclusion); 136 (34.4%) were men; The mean age: 71 ± 8 yrs | SBP, DBP | Age, body mass index (BMI), and antihypertensive medication use, Outdoor temperature, Seasonal variation and standard deviation of room temperature. | The study found that a 1°C increase in room temperature significantly reduced SBP by 0.594 mmHg in the morning (95% CI: -0.644 to -0.545) and 0.680 mmHg in the evening (95% CI: -0.742 to -0.619), with similar reductions in DBP. The association was weaker in July and August but remained significant overall, even after adjusting for confounders. |
| Umishio et al. (2021) | Cross-sectional | Japan | Individuals aged 20 years or older from households intending to perform insulation retrofitting | 3,785 participants from 2,162 households; 1,782 (47%) were men; The mean age: 58 ± 13 yrs | Home blood pressure variability: diurnal variability (ME difference in BP) and day-by-day variability (SD of BP). | Age, sex, BMI, household income, salt intake, vegetable consumption, smoking, alcohol use, exercise, antihypertensive drug use, sleep quality/duration, and number of measurement days, outdoor temperature | Each 1°C increase in ME difference of indoor temperature was associated with a 0.85 mmHg increase in ME difference of systolic BP (95% CI: 0.71 to 0.99, p < 0.001). Each 1°C increase in SD of indoor temperature was associated with a 0.61 mmHg increase in SD of systolic BP (95% CI: 0.47 to 0.75, p < 0.001). No significant association was found between outdoor temperature instability and HBP variability after adjustment |
| Song et al. (2022) | cross-sectional | Six regions in China across three climate zones: Severe Cold Zone (Harbin, Qiqihar), Cold Zone (Dalian, Beijing), and Hot-Summer and Cold-Winter Zone (Nanjing, Shanghai, Changsha, Chengdu), China | Elderly individuals aged 60 and above from urban and rural areas. | Total:  Urban participants: 331 individuals (154 men, 177 women).  Rural participants: 406 individuals (199 men, 207 women).  Indoor temperature monitoring:  89 participants from 65 household;  Urban participants: 35 individuals (19 men, 16 women).  Rural participants: 54 individuals (29 men, 25 women). | Prevalence of hypertension,  SBP, DPB. | Age, BMI, smoking, alcohol consumption, dietary habits (e.g., pickle and deep-fried food consumption), and thermal sensation. | The prevalence of hypertension was approximately 40% in both urban and rural areas. Higher prevalence associated with rural residency (adjusted odds ratio [AOR]: 1.465, 95% CI: 1.063–2.020, p = 0.020)  A 1°C increase in indoor temperature was associated with a decrease in hypertension prevalence (AOR: 0.959, 95% CI: 0.922–0.997, p = 0.036). |
| Li et al. (2023) | Cross-sectional | Shandong Province, China | Rural residents | 46; 22 (47.8%) were men; The mean age: >65 years 72.39 ± 5.90 yrs, ≤65 years 56.70 ± 8.80 yrs | SBP, DBP, pulse pressure difference, heart rate, blood flow velocity, and hand skin temperature. | Age, BMI, clothing Insulation, time of day, heating types and quality. | The study found that colder indoor temperatures significantly increase cardiovascular health risks among the elderly, with systolic blood pressure rising by 1.05 mmHg per 1°C decrease (95% CI: −0.85 to −2.26), heart rate increasing by 0.99 bpm per 1°C decrease (95% CI: −0.97 to −1.00), and the predicted prevalence of hypertension decreasing by 1.9% per 1°C increase, emphasising the importance of maintaining warmer indoor environments to reduce health risks. |
| Kinuta et al. (2023) | Cohort | Masuda City, Japan | Healthy community-dwelling individuals aged 20 to 74 years | 352; 190 (54.0%) were men; The mean age: 49.8 ± 12.2 yrs | SBP, DBP | age, sex, smoking status, alcohol drinking, obesity, physical activity, diabetes mellitus, and antihypertensive medication use. | A 1°C increase in indoor temperature reduced morning SBP by 0.37 mmHg (95% CI: -0.42 to -0.32) and morning DBP by 0.22 mmHg (95% CI: -0.26 to -0.18). Evening SBP and DBP reductions were stronger: 0.45 mmHg (95% CI: -0.51 to -0.40) and 0.30 mmHg (95% CI: -0.35 to -0.26), respectively. The association of BP was stronger for indoor temperature than outdoor temperature. When both were included in the model, the association for indoor temperature persisted while that for outdoor temperature was attenuated |
| Chen et al. (2023) | Cross-sectional | Chengdu, China | Healthy middle-aged and elderly adults aged 50 years and above. | 172 (114 after exclusion); 38 (33.3%) were mean; The mean age: 58.9 ± 7.5 yrs. | SBP, DBP, Blood pressure variability | Age, sex, BMI, salt intake, smoking, alcohol consumption, weekly exercise, and sleep quality. | A 1°C decrease in morning indoor temperature was associated with an increase in SBP by 0.39 mmHg. However, the effect on DBP was not significant.  Morning temperature fluctuations, especially greater than 1.1°C, were found to significantly increase blood pressure variability (BPV), with every 1°C increase in morning temperature fluctuation associated with a 2.18 mmHg increase in SD of SBP. |
| Meade et al. (2024) | Experimental | Ottawa, Canada | Generally healthy community-dwelling older adults (65–80 y), | 16; 10 (62.5%) were man; median 72 yrs (IQR 70–73); | Core body temperature (primary); cardiovascular strain: heart rate, BP, HRV, autonomic function, dehydration | Baseline core temperature; crossover design controlled individual differences | Compared with 22°C, exposure to 31°C and 36°C significantly increased core temperature (+0.7°C [95% CI: 0.5–0.8]; +0.9°C [0.8–1.1]) and heart rate (+9 bpm [6–12]; +13 bpm [10–16]), and reduced SBP (−26 mmHg [−35 to −17]; −29 mmHg [−38 to −20]). HRV indices (e.g., SDNN, RMSSD) declined significantly at ≥31°C. No clinically meaningful changes were observed at 26°C. Findings support ~26°C as an upper indoor temperature limit to minimise cardiovascular and thermal strain. |
| Gonsoroski et al. (2025) | Case–control | New York City, USA | Individuals receiving emergency medical services during summer | Cardiovascular cases n=735; mean age 54 yrs; 50% male | Cardiovascular emergency calls | Outdoor temperature, heat wave status, wind speed (lag 4h), ozone (8-h avg), PM2.5 (24-h avg), age, sex, number of heat-sensitive pre-existing conditions | Indoor temperature ≥28°C was associated with increased odds of cardiovascular emergency calls (OR 1.44; 95% CI: 0.97–2.13), though imprecisely estimated. Outdoor temperature significantly increased cardiovascular risk (OR 1.12; 95% CI: 1.05–1.19). Sensitivity analysis showed no association at ≥26°C threshold. |
| O'Connor et al. (2025) | Panel / repeated-measures observational study | Queensland, Australia | Community-dwelling older adults (≥65 years) | n=47; 15 men (32%); median age 72 year (IQR 68–77) | Nighttime heart rate; HRV indices (lnRMSSD primary; lnHF; lnLF; lnLF:HF) | Individual-level random intercept (mixed-effects model); repeated measures across nighttime hours; secondary analysis excluding heat-sensitive medication use | Compared with <24°C, bedroom temperatures of 24–26°C were associated with higher odds of clinically relevant lnRMSSD reduction (OR 1.4; 95% CI: 1.2–1.6); 26–28°C (OR 2.0; 1.8–2.3); and 28–32°C (OR 2.9; 2.5–3.4) (all P<0.001). Heart rate increased significantly at ≥26°C (e.g., 28–32°C: OR 3.9; 95% CI: 3.5–4.4). Findings indicate progressive autonomic disruption above 24°C, with greater cardiovascular strain at higher temperatures. |

Table S2. Characteristics of exposure assessment

| **Exposure assessment techniques** | **Sensor Types** | **Sensor’s location** | **Monitoring periods and temporal resolution** | **Exposure and exposure range** | **Calibration** | **Integration with Individual Behaviour or housing characteristics** | **Study** |
| --- | --- | --- | --- | --- | --- | --- | --- |
| Indoor monitor | Thermometer | Living room, bedroom 60 cm above the floor | Two 48-hour sessions; 10-minute intervals | Indoor temperature; Spring/fall mean: 20.2 °C to 20.8 °C  Winter mean: 10.3 to 14.1 °C | Not specified in the article | Self-reported home/out-of-home periods | Saeki et al. (2014) |
|  | Temperature and relative humidity monitor (Hobo); | Participant's residence | 8 weeks; 10-min intervals | Indoor temperature;  Mean: 23.49±2.21 °C  Range:  16.71 to 34.25 °C | Download data instruments, zero check, Clean the impaction plates weekly, | Self-reported daily diaries for whether windows were open, use of air conditioner | Goldberg et al. (2015) |
|  | Indoor temperature: Blood pressure monitoring device (Omron HEM-7251G;  Room temperature: Temperature and humidity monitor (TR-72wf T&D) | Living room, bedroom, and changing room | 4 winter periods; Indoor temperature: twice in the morning and twice in the evening.  Room temperature:  10-min intervals | Indoor temperature, Room temperature;  Indoor: morning mean: 14.5±3.4 °C  Range: 3.3 to 25.2℃  Evening mean: 17.8±3.2 °C  Range: 4.3 to 27.5°C | Not specified in the article | Self-reported daily diaries for daily wake-up time, bedtime. | Umishio et al. (2019) |
|  | Digital Thermometer (Digitron 20461) | Living room | Single-point measurement | Indoor Temperature;  The study categorised participants based on their indoor temperature into groups. | Not specified in the article | None | Zhao et al. (2019) |
|  | Electronic hygro-thermograph (AE-817CE) | Participant's residence | 18 days; Twice daily | Indoor temperature;  Morning mean: 29.6°C  Range 24.2 to 38.3°C  Afternoon mean: 31.3°C  Range: 22.9 to 37.3℃ | 20 devices in the same indoor space, measured for 40 min at 15-minute intervals, adjusted based on WBGT levels using QUESTtemp°30. | None | Kim et al. (2020) |
|  | Psychrometer | Not specified in the article | 1 year; Biweekly | Indoor temperature;  N/A | Not specified in the article | None | Ni et al. (2020) |
|  | Blood pressure monitoring device (OMRON HEM-9700T) | Participant's residence | 1 year; Twice daily | Indoor temperature;  Morning mean: 20.0 ± 5.7 °C  Range 2.0 to 36.5 °C  Afternoon mean: 22.4 ± 4.9 °C  Range: 2.0 to 37.0℃ | Compared the temperature obtained by the sphygmomanometer with that of a thermometer (BM-727, Empex instruments, Tokyo, Japan) by changing the temperature 20 times. | None | Kubozono et al. (2021) |
|  | Temperature and humidity monitor (TR-72wf T&D) | living room, 1.0 meter above the floor | 2-week; 10-min intervals | Indoor temperature;  Morning-Evening mean: 16.3 ± 3.1 ℃  Range: 4.5 to 25.6 ℃  The mean Morning-Evening difference in indoor: 3.2 ℃ | Not specified in the article | Self-reported daily time spent at home | Umishio et al. (2021) |
|  | Room temperature: liquid crystal thermometers.  Continuous indoor and outdoor temperature and humidity: thermometer (TR-76Ui) | Living room, bedroom and toilet, 1 m above the floor near the internal wall. | 1-week; temporal resolution not specified | Indoor temperature;  5°C to 26°C | Not specified in the article | Questionnaire about residents' daily time spent outside and housing characteristics | Song et al. (2022) |
|  | Temperature recorder (TR-72NW T&D). | Participant's residence, 1.5 metres height near the participants | Four-month; 1-min intervals | Indoor temperature;  N/A | Not specified in the article | None | Li et al. (2023) |
|  | Blood pressure monitoring device (OMRON HEM-9700T) | Participant's residence | Two-years; Twice daily | Indoor temperature;  Indoor Temperature: Morning: 19.0°C (IQR: 15.0°C–24.0°C); Evening: 21.0°C (IQR: 18.0°C–25.0°C) | Same types of devices verified by pervious study | None | Kinuta et al. (2023) |
|  | Temperature recorder (Tian Jian Hua Yi WWZY-1) | Living room and bedroom, at a height of 70 cm above the ground | 3-months cold winter and 3-months mild spring and autumn; 10-min intervals | Indoor temperature and temperature fluctuation;  Morning mean temperature: 15.3 ± 3.3 °C  Sleeping mean temperature: 15.4 ± 3.3 °C | Not specified in the article | None | Chen et al. (2023) |
|  | SHT45 temperature & humidity sensors | Bedroom; mounted 1.1–1.7 m above floor on internal wall, away from solar radiation and appliances | Summer season (Dec–Mar); 10-minute intervals; |  | Manufacturer accuracy ±0.1 °C; artefact removal (<5% threshold); quality control applied | None | O'Connor et al. (2025) |
| Temperature controlled room | N/A | N/A | Short term | Indoor temperature;  Indoor temperature changes of ±10°C (18°C to 28°C or vice versa) for 20 minutes in controlled settings. | Not applicable | None | Chen et al. (2021) |
|  | N/A | N/A | Four randomised 8-h indoor exposure sessions | Indoor temperature conditions: 22°C, 26°C, 31°C, 36°C (45% RH); simulated overheating exposure | Not applicable | None | Meade et al. (2024) |
| Portable monitor | Temperature and humidity (HOBO U23 Pro v2 sensors) | Inside the buildings where patients received emergency medical care | Short term; 2-min intervals | Indoor temperature;  Daytime mean: 23.9 ± 2.5 °C  Range: 13.5 to 33.5 °C  Night-time mean: 22.9 ± 2.5 °C  Range: 12.8 to 29.3 °C | Not specified in the article | None | Uejio et al. (2016) |
|  | Temperature and humidity (HOBO U23 Pro v2 sensors) | Inside the buildings where patients received emergency medical care | Short term; 3-min intervals | Indoor temperature measured; range 13.16–35.30 °C; high exposure defined as ≥28 °C | Removal of spurious readings; cross-sensor comparison and outlier inspection; anomalous values removed | None | Gonsoroski et al. (2025) |
| Modelling | Estimated by a prediction model based on hourly indoor temperature data collected from a previous study | None | Two years; Hourly | Cumulative Degree Hours (CDH): CDH were calculated using the estimated indoor temperatures for both the hot (May to October) and cold (November to April) seasons; | Not applicable | Prediction model incorporated building characteristics | Jung et al. (2020) |
